# Microarray Expression Profiling and Raman Spectroscopy Reveal Anti-Fatty Liver Action of Berberine in a Diet-Induced Larval Zebrafish Model

**DOI:** 10.3389/fphar.2019.01504

**Published:** 2020-01-08

**Authors:** Bo Chen, Yang-Min Zheng, Miao-Qing Zhang, Ying Han, Jing-Pu Zhang, Chang-Qin Hu

**Affiliations:** ^1^ Key Laboratory of Biotechnology of Antibiotics, The National Health Commission (NHC), Institute of Medicinal Biotechnology, Chinese Academy of Medical Sciences & Peking Union Medical College, Beijing, China; ^2^ Beijing Key Laboratory of Antimicrobial Agents, Institute of Medicinal Biotechnology, Chinese Academy of Medical Sciences & Peking Union Medical College, Beijing, China; ^3^ Institute of Cerebrovascular Disease Research, Xuanwu Hospital of Capital Medical University, Beijing, China; ^4^ Postdoctoral Scientific Research Workstation, China Resources Sanjiu Medical & Pharmaceutical Co., Ltd., Shenzhen, China; ^5^ Postdoctoral Mobile Research Station, Institute of Process Engineering, Chinese Academy of Sciences, Beijing, China; ^6^ National Institutes for Food and Drug Control, Graduate School of Peking Union Medical College, Beijing, China

**Keywords:** berberine, non-alcohol fatty liver disease, Raman mapping, larval zebrafish, transcriptomics

## Abstract

**Background:** The prevalence of non-alcohol fatty liver disease (NAFLD) is increasing in children and adolescents who are mostly resulted from overfeeding. Previous studies demonstrate that berberine (BBR), a compound derived from plant, has beneficial effects on NAFLD in adults but poorly understood in the pediatric population. This study employed a larval zebrafish model to mimic the therapeutic effects of BBR in the pediatric population and the mechanisms underlying its hepatoprotection.

**Methods:** High-cholesterol diet (HCD)-fed zebrafish exposed to BBR at doses of 0, 1, 5, and 25 μM. After the larvae were treated with BBR for 10 days, its effect on hepatic steatosis was evaluated. We introduced Raman imaging and three-dimensional (3D) molecular imaging to detect changes in the biochemical composition and reactive oxygen species (ROS) levels of zebrafish liver. Gene expression microarray was performed to identify differentially expressed genes (DEGs) followed by gene ontology (GO), Kyoto Encyclopedia of Genes and Genomes (KEGG) pathway, and functional category analysis.

**Results:** BBR (5 and 25 μM) administration prevented HCD-induced liver lipid accumulation in larval zebrafish. The result was further confirmed by the pathological observation. Raman mapping indicated that the biochemical composition in the liver of BBR-treated group shifted to the control. The quantitative analysis of 3D imaging showed that the ROS level was significantly decreased in the liver of BBR-treated larvae. In the livers of the BBR group, we found 468 DEGs, including 172 genes with upregulated expression and 296 genes with downregulated expression. Besides, GO enrichment, KEGG pathway, and functional category analysis showed that various processes related to glucolipid metabolism, immune response, DNA damage and repair, and iron were significantly enriched with DEGs. The expression levels of the crucial genes from the functional analysis were also confirmed by quantitative PCR (qPCR).

**Conclusion:** BBR can significantly improve hepatic steatosis in HCD-fed zebrafish larvae. Its mechanisms might be associated with the regulation of lipid metabolism, oxidative stress, and iron homeostasis. Raman imaging in larval zebrafish might become a useful tool for drug evaluation. Mainly, the gene expression profiles provide molecular information for BBR on the prevention and treatment of pediatric NAFLD.

## Introduction

Non-alcohol fatty liver disease (NAFLD) is characterized by excessive fat accumulation in liver cells, which is cumulatively prevalent due to the worldwide obesity epidemic (Wruck et al., 2017). NAFLD is a risk factor of metabolic diseases, such as type 2 diabetes, obesity, and dyslipidemia (Mahfood Haddad et al., 2017). Indeed, NAFLD can progress to its more severe form non-alcoholic steatohepatitis (NASH) (Day and James, 1998), fibrosis, and may eventually develop into hepatocellular carcinoma (Day, 2006). In general, NAFLD was considered a disease of adults. However, the prevalence of NAFLD is rapidly increasing in children and adolescents, especially in obese children (Hatton et al., 2018). The treatment approach is limited because of unclear pathogenesis of NAFLD and lack of therapeutic agents. Therefore, it is reasonable to develop suitable treatment strategies for children suffering from this disease (Betancourt-Garcia et al., 2017; Assuncao et al., 2017).

Berberine (BBR) is an isoquinoline alkaloid, source of berberis, and has been widely used in traditional Chinese medicine for hundreds of years. Modern pharmacology demonstrates that BBR has antibacterial, anti-diabetic, anti-hyperlipidemic, and multiple cardioprotective effects (Imenshahidi and Hosseinzadeh, 2019). Many studies showed that BBR could regulate metabolism disorder, increase insulin sensitivity, and improve glycometabolism, which may indicate it has potential in the treatment of NAFLD (Zhu et al., 2016). However, most of the animal experiments and clinical trials of BBR were executed in adult animals and the adult population. The histological features of NAFLD/NASH in children and adults are quite different (Nobili et al., 2006). Childhood NASH exhibits a much more common predominance of portal inflammation than in adult NASH, yet the differences of the mechanisms between adults and children in the pathogenesis of NAFLD are poorly understood (Nobili et al., 2016; Hatton et al., 2018). On the other hand, there is a difference in sensitivity to medications between children and adults (Assuncao et al., 2017). So, it is necessary to explore the effects and mechanisms of BBR on NAFLD in children and young animal models.

Zebrafish are a small vertebrate organism that has been applied for *in vivo* models in various fields including genetics, developmental biology, toxicology, and preclinical medicine experiments (Barros et al., 2008; Albadri et al., 2017; Chen et al., 2017). Zebrafish are easy to breed and mature fast as well as cost little in daily maintenance. Recent studies demonstrated that zebrafish has a similar process with humans in lipid metabolism (Holtta-Vuori et al., 2010). Moreover, because the zebrafish larvae have optical transparency, the livers can be easily observed in real time (Schlegel, 2012). Recently, zebrafish have become increasingly popular in research of pediatric diseases, such as acute leukemia models and infectious diseases (Veldman and Lin, 2008; Lohi et al., 2013). Several zebrafish models in both adults and larvae have been well established (Gu et al., 2014; Sapp et al., 2014; Dai et al., 2015; Chen et al., 2018) to study NAFLD. That makes it possible to evaluate the drugs in the larvae. Zebrafish have therefore been used as alternatives to rodents for animal models in the present study.

Herein, we constructed diet-induced steatosis in zebrafish larvae that could mimic human children's disease. In particular, the role of BBR on NAFLD in the larval zebrafish model remains to be determined. This study aimed to evaluate the effects and mechanisms of BBR on hepatic steatosis and provide data support for BBR in the treatment of pediatric disease. To get further insights into the molecular mechanism for the anti-fatty liver action of BBR, we performed microarray analysis to define the differences in the transcriptome of liver from diet-induced zebrafish larvae treated with or without BBR and validated the expression levels of the crucially differentially expressed genes (DEGs) that were identified by functional category analysis. Furthermore, we introduced Raman confocal microspectroscopy and three-dimensional (3D) molecular imaging to analyze the changes of biochemistry component and reactive oxygen species (ROS) levels in the larval liver, respectively.

## Methods and Materials

### Ethical Approval

This study was reviewed and approved by the Laboratory Animal Management and Animal Welfare Committee in the Institute of Medicinal Biotechnology, Chinese Academy of Medical Sciences, and we always made the most effort to minimize animals' suffering.

### Zebrafish Care and Treatment

AB strain zebrafish (*Danio rerio*) and Tg (fabp10a: dsRed) zebrafish were kept under standard laboratory conditions with a 14-h light/10-h dark cycle at a temperature of 28.5 ± 1°C (Hu et al., 2011). Embryos and larvae were maintained in embryo medium to 5 days post-fertilization (dpf) without feeding. Zebrafish larvae were fed with control diet (AP100, 30 mg/100 fish/day) or a high-cholesterol diet (HCD, 180 mg AP100 plus 4% cholesterol/100 fish/day) for 10 days. For hepatic steatosis analysis, larvae in the HCD group were exposed to BBR at doses of 0, 1, 5, and 25 μM for a 10-day treatment period until 15 dpf. The doses selected in this study were referred to the ones used in previous studies (Zheng et al., 2018). According to the results of hepatic steatosis analysis, 5 μM of BBR was applied to the subsequent experiments, including histologic analysis, Raman measurement, ROS detection, microarray analysis, and quantitative PCR (qPCR).

### Whole-Mount ORO Staining

Zebrafish larvae were fixed in 4% paraformaldehyde (PFA) overnight at 4°C and washed twice with phosphate-buffered saline (PBS). Oil Red O (ORO) staining was performed as described before (Sapp et al., 2014). Lipid droplets in liver tissue were observed and imaged on a bright-field dissecting microscope (Olympus szx10, Tokyo, Japan). Larvae were defined as positive for steatosis according to the previous report (Sapp et al., 2014).

### Histologic Analysis

Larvae were fixed with 4% PFA overnight, coated in paraffin, and cut into 4-μm slices with paraffin microtome. The sections were stained with hematoxylin and eosin (H&E). For ORO staining of cryosections, larvae were embedded in OCT (Sakura Japan Co., Ltd., Tokyo, Japan) and stored at −80°C until sectioning. Serial sections (8 μm) were cut. Each section was stained with 0.5% ORO. Slides were observed using an Olympus BX53 microscope (Olympus, Tokyo, Japan). The positive area of ORO was calculated with ImageJ software.

### Electron Microscopy

After the experiment, zebrafish larvae were collected and fixed in buffered 2% glutaraldehyde. Briefly, the fixed larvae were mounted and sectioned. Then the slides were imaged by a transmission electron microscope (Tecnai Spirit, FEI, USA) as in previous studies (EauClaire et al., 2012).

### Biological Analysis

Levels of total cholesterols (TCs) and triglycerides (TGs) of the dissected liver of zebrafish larvae were determined by Total Cholesterol Reagent Kit and Triglyceride Reagent Kit (Applygen Technologies Inc., Beijing, China) according to the manufacturer's specification, respectively. The glutathione (GSH) and malondialdehyde (MDA) levels of zebrafish tissue were determined by using the respective reagent kits according to the manufacturer's specification (Nanjing Jiancheng Biotechnology Institute, Nanjing, China).

### Raman Measurements of Liver Tissues

Raman mapping was performed by using a 780-nm wavelength excitation laser with a ×50-μm pinhole for confocality (DXRxi Raman imaging microscope, Thermo Fisher Scientific Inc., Hudson, USA). The laser power and accumulation time were set at 10 mW and 60 s, respectively. Raman spectra were collected over a scanning spectral range of 600–3,200 cm^−1^. Each liver of zebrafish was determined under the microscope at least three different regions for at least three times to acquire the average spectrum. System control and spectrum acquisition were conducted using OMNIC software (Thermo Fisher Scientific Inc., Hudson, USA). Zebrafish larvae were anesthetized with tricaine (Sigma, USA) before the Raman experiment.

### 3D Imaging to Detect ROS Level and Macrophage of the Whole Liver

Live Tg (fabp10a: dsRed) zebrafish were incubated with CellROX^®^ Green (ThermoFisher, USA), a ROS sensor, at a final concentration of 10 μM for 1 h in the dark, washed in PBS twice, anesthetized using tricaine (Sigma, USA), and fixed with PFA. For macrophage detection, larvae were incubated with the L-plastin antibody (Santa Cruz, USA, 1:500 dilution) at 4°C overnight, and then with an Alexa Fluor488-conjugated antibody (ZSGB-BIO, Beijing, China, 1:1,000 dilution) at room temperature for 2 h. Then the fish liver was isolated and placed upon the ×10 detection lens of the Deltavision wide-field deconvolution imaging system (DV; GE Healthcare, USA). After image acquisition, a 3D view of the liver was generated from original data for quantitative analysis by using softWoRx (GE Healthcare, USA). All objects of interest were collected and quantified during the construction of this 3D model.

### Microarray Expression Profiling and Bioinformatic Analysis

Total RNA was extracted using TAKARA RNAiso Plus according to the manufacturer's instructions, followed by RNA quality evaluation by Agilent Bioanalyzer 2100 (Agilent Technologies, Santa Clara, CA, USA). The Agilent zebrafish (V3) gene expression microarray 4×44K (Design ID 026437) was used for microarray analysis. The process of microarray scanning was carried out by CapitalBio Corporation (Beijing, China) as previously described (Han et al., 2018). To identify DEGs between the control, HCD, and BBR-treated group, the threshold of fold change ≥2 was established in our analysis. Then, based on the KEGG and GO databases, biomedical pathways were classified (Kanehisa and Goto, 2000). Functional category analysis was performed by the Database for Annotation, Visualization, and Integrated Discovery (DAVID) bioinformatics program (http://david.abcc.ncifcrf.gov/) under a significance threshold of *P* < 0.05. We used the Cytoscape software to map the gene–pathway interaction network (http://www.cytoscape.org/), the shinyCircos software to map the Circos plot (Yu et al., 2018), and TBtools software to map the heatmap plot (https://www.biorxiv.org/content/10.1101/289660v1). The microarray data were deposited in Gene Expression Omnibus (GEO accession: GSE136436).

### Quantitative RT-PCR

Total RNA was isolated from dissected livers of zebrafish larvae by TRIzol reagent (no.15596026, Thermo Fisher, USA) and then converted into complementary DNA (cDNA) by reverse transcriptase (no. M1701, Promega, USA). The cDNAs were then used as the templates for quantitative real-time polymerase chain reaction (qPCR). The reaction of qPCR was performed by the real-time quantification system (ROCHE LightCycler 96, Switzerland). The relative amount of messenger RNAs (mRNAs) was calculated with β-actin mRNA as the invariant control. The relative transcript expression level was determined using the control sample as a calibrator and the ^ΔΔ^CT method. The following specific primers were used for amplification in this study (**Table 1**).

**Table 1:** Primer for qPCR genes used in this study.

Gene symbol	Sense primer (5′–3′)	Antisense primer (5′–3′)
*jmjd6*	CTCGTCCTCATCCTCCTC	ATGCCTCCTGTTCTCCTC
*isca1*	GTCAGATGAGGAGGTGTTAC	CCTTGATGTTGGGATTGTTG
*ch25h*	TGGCATCTTCTACATCAC	GAGCATCTCTGTCATAGG
*fads2*	CCATCGGCACTTCCAGCATCAC	TTTCCCACCACAAAGGCGTTCAG
*mvda*	CTGAACAAGTGGCATCTG	CCTTATCATCTCTTCCATACG
*plcg1*	AAGCAGAGAAGTATGTGAAC	GTCGTAGTTGGAGGAGTC
*h2afx*	TGTTCACCGTCTCCTTCG	AGTCTTCTTGTTGTCTCTTGC
*pif1*	AGCCATAGCCAGGTCCATC	ATACACTTCCGCCAACTTCG
*primpol*	ATGAGCCACTTACTGATG	CTCCTCTTCTTCTACTTCG
*ube2t*	GTTCTCACATCCATACAGTTATTG	AGTCTTTCCATCCGTCTCC
*tk1*	CCAGACACCGTTGAGTTC	ACCAGATTCAGGATGTTACC
*lpl*	ATGGAATACACGGCGAGAAG	CAGTTTGCGAATGTGGAAGG
*parpbp*	TTGGATGAGGAGGCATTAGC	TGGACTTGATATGTTCTTGTTGG
*rev1*	GCTCCAAGTTGACCTCTC	CGAATGTTGTAGTTCATCTCC
*msmo1*	GTTGTGCTGTAATTGAAGAC	GGTGCCAATGAAGAATCC
*βactin*	CCGTGACATCAAGGAGAAG	ATACCGCAAGATTCCATACC

### Statistical Analysis

Data are expressed as means ± SEM. Comparisons between multiple groups were performed using one-way ANOVA with Tukey's *post hoc* test. In all experiments, differences were considered statistically significant for *P* < 0.05.

## Results

### The Effects of BBR on Body Weight and Body Length in HCD Diet-Fed Zebrafish Larvae

**Figure 1:**
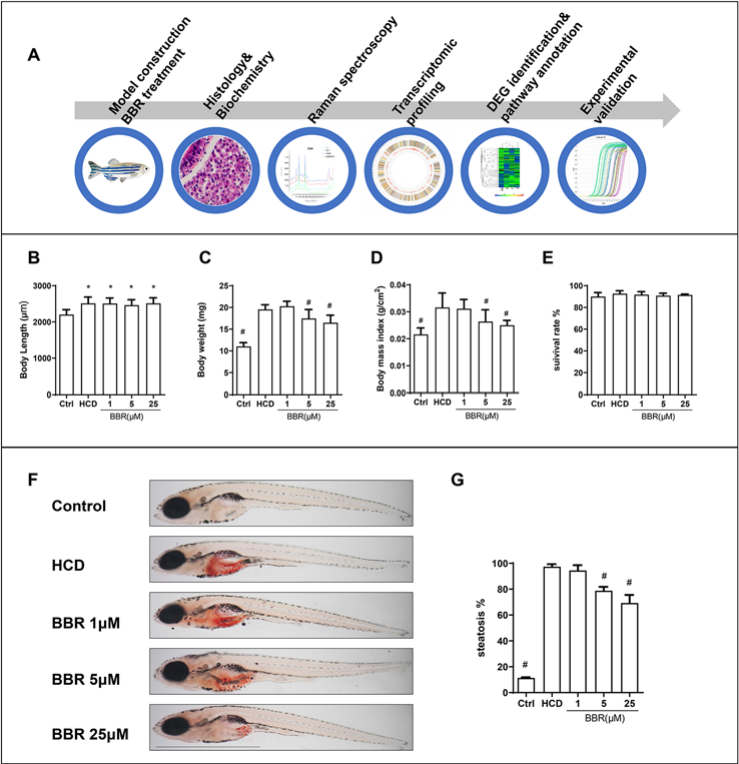
Effects of high-cholesterol diet (HCD) and berberine (BBR) treatment on the growth of zebrafish. **(A)** Description of the experimental design. **(B)** Body length, **(C)** average body weight, and **(D)** BMI in each group were measured at 15 days post-fertilization (dpf). **(E)** Larvae with different treatments were scored for mortality. **(F)** The hepatic steatosis of zebrafish was detected by whole-body Oil Red O (ORO) staining. **(G)** Percent of larvae with hepatic steatosis. *Compared with the control; ^#^compared with the HCD group, *P* < 0.05. *Bar*, 1 mm.

Embryos and larvae were maintained in embryo medium to 5 dpf, and zebrafish larvae without malformation were divided into five groups randomly. For model building, 5-dpf zebrafish were treated with control (30 mg AP100/100 fish/day) or HCD (180 mg AP100 plus 4% cholesterol/100 fish/day) for 10 days. HCD diet-fed zebrafish were exposed to different doses of BBR (0, 1, 5, and 25 μM). After 10 days of feeding and treatment, the zebrafish were washed and collected for the following experiments. The main experimental design was graphed as **Figure 1A**. We firstly did general observation and measured the larvae body and length. All the BBR-treated zebrafish larvae did not exhibit obvious abnormalities and varying mortality (**Figure 1E**). Compared with the control group, the body length in other groups were significantly larger (**Figure 1B**). The body weight was significantly heavier in the HCD group than those in the control group (**Figure 1C**). Meanwhile, the HCD diet-fed larvae had a higher body mass index (BMI) than the control group (**Figures 1C**, **D**). Particularly, BBR treatment significantly decreased body weight and BMI, but did not affect body length (**Figures 1B–D**).

### BBR Attenuated HCD Diet-Induced Hepatic Steatosis in Larval Zebrafish

**Figure 2:**
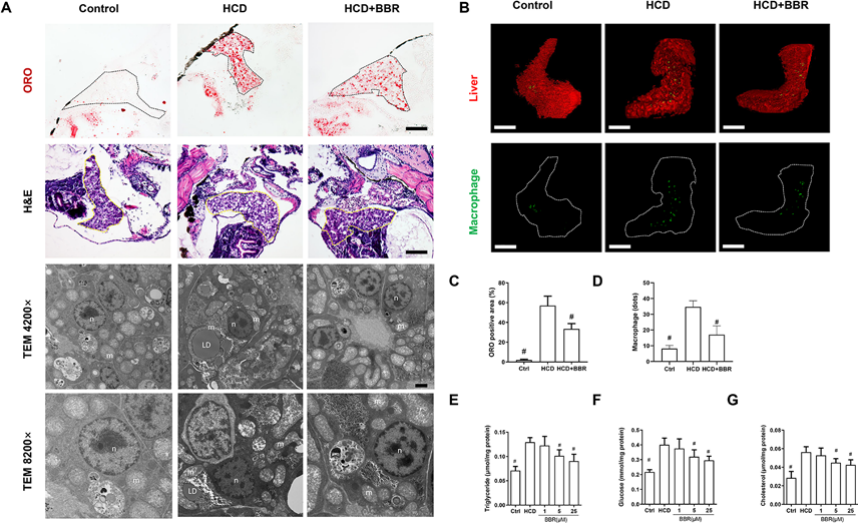
Effects of berberine (BBR) on hepatic steatosis in high-cholesterol diet (HCD)-induced zebrafish larvae. **(A)** The zebrafish larvae were treated with the control diet, HCD diet, and HCD plus BBR (5 μM) treatment. Hepatic steatosis was observed by whole-body Oil Red O (ORO) staining (line 1, refer to the black dotted, bar = 100 μm), H&E staining (line 2, refer to the yellow dotted, bar = 100 μm), and TEM (line 3, bar = 2μm; line 4, bar = 1 μm). *m* mitochondrion, *n* cell nucleus, *LD* lipid droplet. **(B)** L-plastin-labeled macrophages in Tg (fabp10a: dsRed) zebrafish. The statistical results of the positive area of ORO staining were shown in **(C)** and the quantity of macrophage in the liver of larval zebrafish in **(D)**. Effects of BBR on the levels of triglyceride **(E)**, glucose **(F)**, and cholesterol **(G)** in livers of zebrafish larvae fed with HCD. ^#^Compared with the HCD group, *P* < 0.05.

We then assess the effects of BBR on liver lipid accumulation in zebrafish. The whole body of zebrafish larvae was stained with ORO. HCD diet-induced significant steatosis in the liver and the incidence of steatosis in the model group were much higher than in the control group (**Figure 1F**). Larvae treated with BBR (5 and 25 μM) observably decreased the incidence of steatosis compared with the HCD group (**Figure 1G**). The results of the frozen liver section with ORO staining further confirmed the effects. The relative staining area of ORO in the model group was higher than in control. BBR treatment significantly reduced the ORO positive compared with the model group (**Figures 2A**, line 1, and **C**). The paraffin section with H&E staining showed severe macrovesicular steatosis in the liver of HCD diet-fed zebrafish, and the liver lesion was much improved by BBR treatment (**Figure 2A**, line 2). Transmission electron microscopy (TEM) was employed to observe the change of hepatocyte in zebrafish larvae. Several large sizes of lipid droplets were found in the HCD group, but not in the control group. Mainly, we noticed the changes in the mitochondria morphology in the HCD group, including mitochondrial elongation, tubular, and crest broke. BBR treatment rescued HCD-induced mitochondrial damage in the hepatocyte of larval zebrafish (**Figure 2A**, lines 3 and 4). Then the TC, TG, and glucose levels in the isolated liver from zebrafish were carefully measured. The levels of TC, TG, and glucose were notably elevated in the liver of the model group compared with the control group, and HCD-fed zebrafish with BBR treatment significantly reduced the hepatic TC, TG, and glucose contents (**Figures 2E**–**G**). To address the inflammatory response in larvae with HCD, we used the L-plastin antibody to label macrophage (Stoletov et al., 2009). The results showed that a small amount of hepatic macrophage was present in the liver of control larvae, and the number of macrophages were increased in the HCD group. BBR treatment could reverse the effect of HCD on macrophage recruitment to the liver in zebrafish (**Figures 2B**, **D**). Furthermore, Sirius red staining was employed to assess the development of fibrosis, but no significant fibrosis was found in the three groups (**Figure S1**). The above results demonstrated that BBR could significantly improve diet-induced liver steatosis and exert a protective effect on hepatocyte in larval zebrafish.

### Raman Spectroscopy Analysis

**Figure 3:**
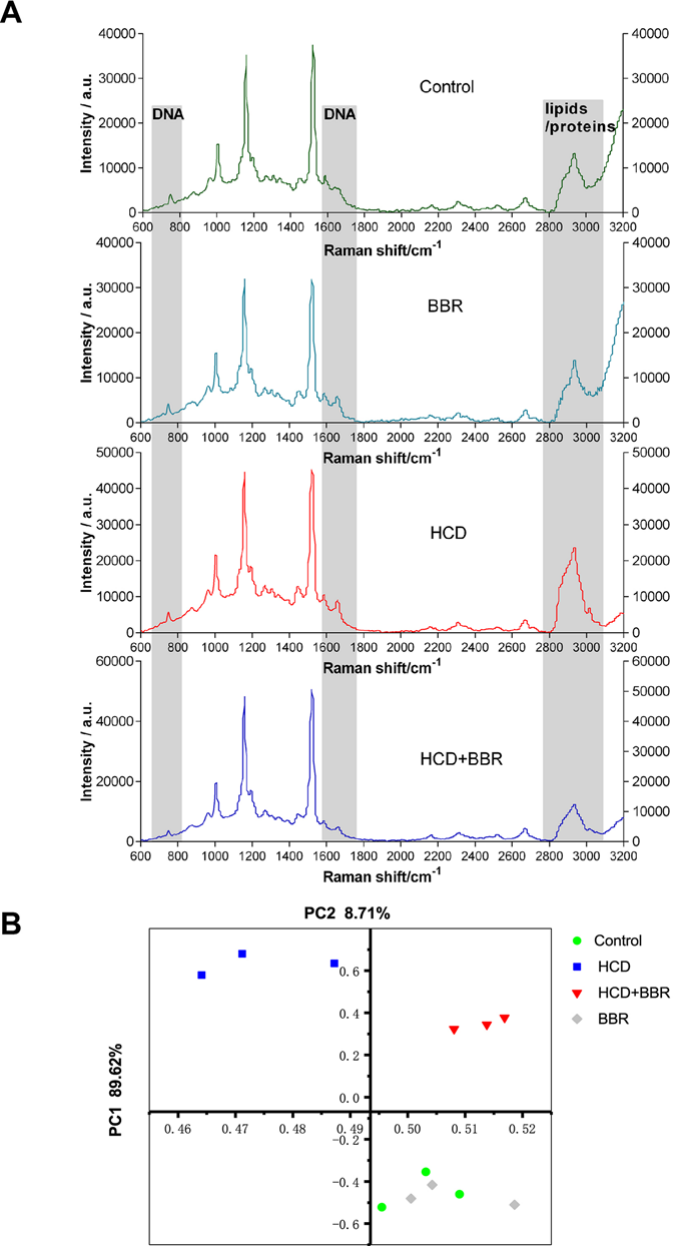
Raman spectral analysis of liver tissue of zebrafish larvae treated with berberine (BBR). **(A)** Raman spectra obtained from the liver of zebrafish with different treatments. **(B)** Principal component analysis (PCA) of the spectra extracted from the Raman spectral datasets of the control, BRR(5 μM), HCD, and HCD+BBR (5 μM). Each liver of zebrafish was determined under the microscope at least three different regions for at least three times to acquire the average spectrum.

Raman mapping of individual liver regions of zebrafish embryos at a microresolution level was applied to provide information on their biochemical composition (**Figure 3**). The Raman spectra obtained from the liver of different groups exhibit differential spectral lines at around 720–790, 1,660, and 2,800–3,025 cm^−1^ (**Figure 3A**). The Raman maps using the C–H stretching mode in the 2,800- to 3,025-cm^−1^ region were associated with lipids and proteins (Majzner et al., 2014). Besides, DNA peaks can be demonstrated at 722, 752, 782, and 1,663 cm^−1^ (Shetty et al., 2006). Compared with the control liver, the spectra of regions at 1,663 and 2,800–3,025 cm^−1^ showed higher intensity in the HCD group, demonstrating an increased distribution of lipid and DNA in the liver. The results showed that BBR (5 μM) had the ability to modulate liver lipid, DNA, and protein. Raman signal intensity revealed that the levels of lipid and DNA were decreased in the liver of zebrafish treated with BBR (**Figure 3A**). The principal component analysis (PCA) indicated the significant alterations in the biochemical composition of liver between the control and HCD groups, while the scatter plots could shift to the control group by BBR treatment, suggesting that BBR treatment has liver protection against HCD in larval zebrafish (**Figure 3B**). This approach further confirmed the histological and biochemical results and provided more detailed information on macromolecular composition.

### Quantification Analysis of Hepatic ROS Level Based on 3D Models of the Whole Liver

**Figure 4:**
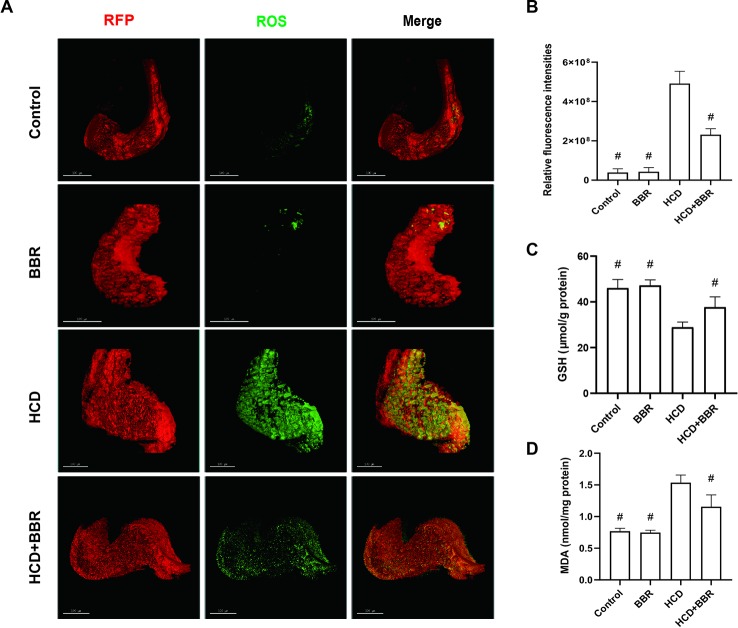
Effects of berberine (BBR) on the hepatic oxidative stress in high-cholesterol diet (HCD)-induced zebrafish larvae. **(A)** Liver volume rendering of reactive oxygen species (ROS) staining showed that BBR (5 μM) attenuated hepatic ROS level in HCD-induced Tg (fabp10a: dsRed) zebrafish. ROS generation in the Tg (fabp10a: dsRed) zebrafish liver treated with HCD. Hepatic ROS were detected with CellROX^®^ Green Reagent. Bar = 100 μm. **(B)** Quantitative analysis of ROS throughout the entire zebrafish liver. Effects of BBR (5 μM) on the glutathione (GSH) **(C)** and malondialdehyde (MDA) **(D)** levels in HCD-induced zebrafish larvae. ^#^Compared with the HCD group, *P* < 0.05.

Oxidative stress promotes the progression of NAFLD in the form of NASH (Richter and Kietzmann, 2016). The increase of cellular ROS generation results in organelle and cellular dysfunction, and apoptosis ultimately (Paradies et al., 2014; Spahis et al., 2017). Here, we introduced a method for quantitative analysis and 3D imaging, which use deconvolution algorithms, rapidly and precisely reconstructing 3D objects, allowing a better visual understanding of the image data and a more accurate quantitative assessment of the 3D object (Pegard and Fleischer, 2013; Chakrova et al., 2016). Compared with the control group, the HCD feeding significantly induced the hepatic ROS levels increase, which was inhibited by BBR (5 μM). While BBR treatment alone did not affect the ROS generation (**Figure 4A**). MDA levels were significantly higher in the HCD-treated groups, whereas the GSH level was significantly lower. BBR treatment could induce the increase of GSH level and inhibit the production of MDA. No apparent changes were observed in the 5-μM BBR-treated group (**Figures 4C, D**). These results suggested that BBR could alleviate the oxidative stress in HCD-induced zebrafish and do not affect the ROS balance in normal larval zebrafish.

### Global Changes in Gene Expression of Larval Zebrafish Liver

**Figure 5:**
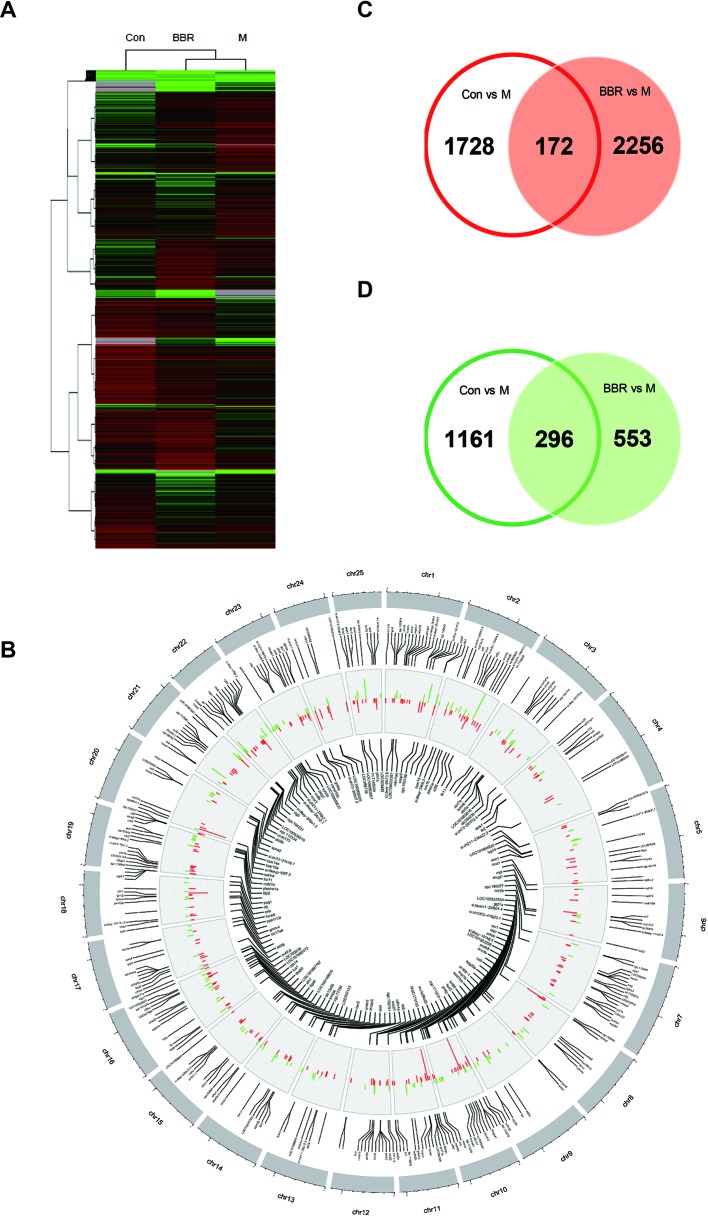
Trend analysis of gene expression patterns in larval zebrafish liver. **(A)** Cluster analysis of genes and samples from differentially expressed genes (DEGs). **(B)** The common DEGs in control and BBR groups compared with the HCD group shown by Circos plot. Red color, upregulated genes; green color, downregulated genes. **(C)** Comparison of upregulated DEGs between different groups. **(D)** Comparison of downregulated DEGs between different groups.

After 10 days of treatment, the livers of zebrafish larvae (control, HCD, and BBR 5 μM) were applied to microarray analysis. The data of gene expression were obtained by using the zebrafish gene expression 4 × 44K microarray (Agilent). The array data were analyzed for data summarization, normalization, and quality control by using the GeneSpring software V13 (Agilent). To investigate the response to the HCD and BBR treatment, we performed cluster analysis. The results indicated that the BBR groups displayed closer expression patterns than the HCD groups compared to the control group. The threshold values of ≥2 and −2-fold change or less and Benjamini–Hochberg corrected *P* value <0.05 were selected as the differentially expressed genes. Compared to the HCD (shortened to M in **Figure 5**) group, 3,357 and 3,277 DEGs were identified in the control group and BBR group, respectively (**Figure 5A**). The DEGs with log2 (Ctrl vs. M) > 1 and log2 (BBR vs. M) > 1 expression levels and the DEGs with log2 (Ctrl vs. M) less than −1 and log2 (BBR vs. M) less than −1 expression levels at *P* < 0.05 were combined, which leads to the identification of 468 genes with significantly different expression levels in the livers of the BBR group (**Figure 5B**). Of these genes, 172 had increased expression levels and 296 had significantly decreased expression levels (**Figures 5C, D**).

### KEGG Pathway and GO Enrichment Analysis Based on Combined DEGs

**Figure 6:**
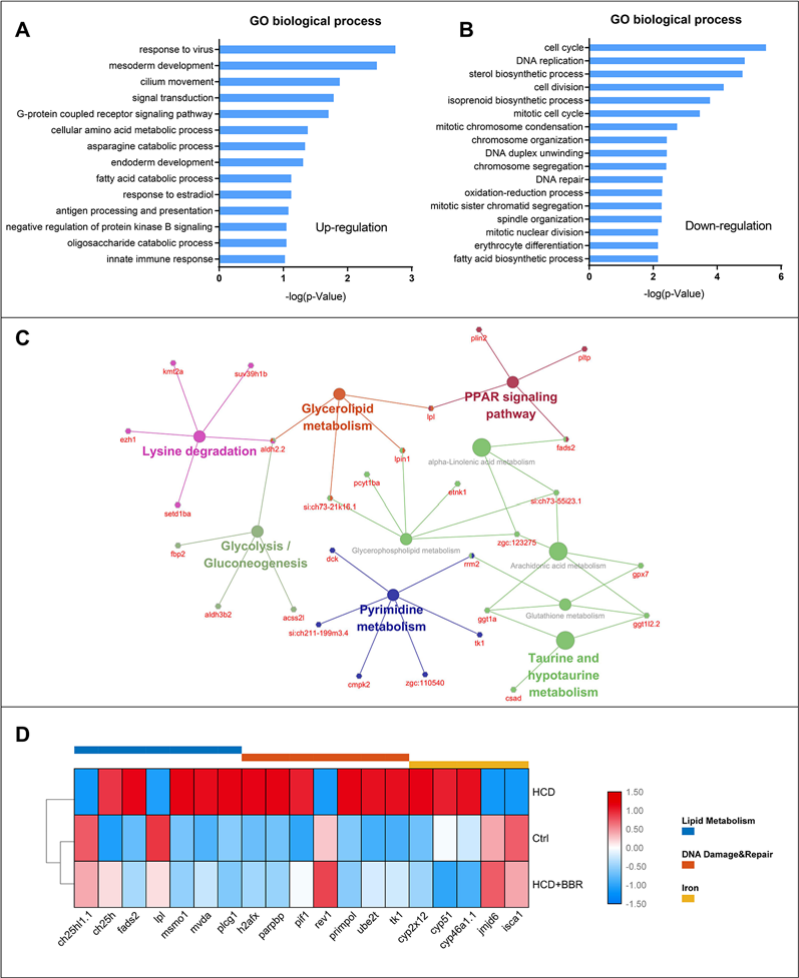
Gene ontology, KEGG enrichment, and functional category analysis. **(A)** Gene ontology from the upregulated differentially expressed genes (DEGs) (Ctrl vs. M combined 5 μM BBR vs. M). **(B)** Gene ontology from the downregulated DEGs (Ctrl vs. M combined 5 μM BBR vs. M). **(C)** KEGG pathway enrichment results for the total DEGs. **(D)** Gene collection from functional category analysis by DAVID.

To figure out the possible mechanism of BBR on the zebrafish NAFLD model, we conducted a function–annotations analysis from the co-DEGs (Ctrl vs. M combined BBR vs. M). The co-upregulation and co-downregulation in DEGs were enriched into the GO biological process, as shown in **Figures 6A**, **B**, respectively. We further applied the KEGG ontology to classify the functional annotation of genes. A total of 124 genes can be assigned to the 10 pathways. As expected, most genes were classified into the metabolic pathways, such as PPAR signaling pathway, glycolysis/gluconeogenesis, glycerophospholipid metabolism, glycerolipid metabolism, and pyrimidine metabolism. We noted that *ggt1a*, *ggt1l2*.2, *gpx7*, and *rrm2* genes were classified into glutathione metabolism term, which may partially explain the antioxidant mechanism of BBR. The gene–pathway interaction network was shown in **Figure 6C**. Functional category analysis by DAVID included lipid metabolism, DNA damage and repair, and iron, which was closely associated with pediatric NAFLD (Feldman et al., 2015; Mann et al., 2017; Baker and Friedman, 2018). Several vital genes involved in these pathways were enriched, including *ch25h1.1*, *ch25h*, *fads2*, *lpl*, *mvda*, *plcg1*, *h2afx*, *parpbp*, *pif1*, *rev1*, *primpol*, *ube2t*, *tk1*, *cyp2x12*, *cyp51*, *cyp46a1.1*, *jmjd6*, and *isca1* (**Figure 6D**). Excessive triglyceride accumulation in the hepatocytes causes oxidative stress, lipotoxicity, and subsequent DNA damage (Mann et al., 2017). These results indicated that the protective effects of BBR on hepatocytes might be related to the improvement of lipid metabolism, oxidative stress, and iron homeostasis.

### Validation of Microarray Analysis Genes by qRT-PCR

**Figure 7:**
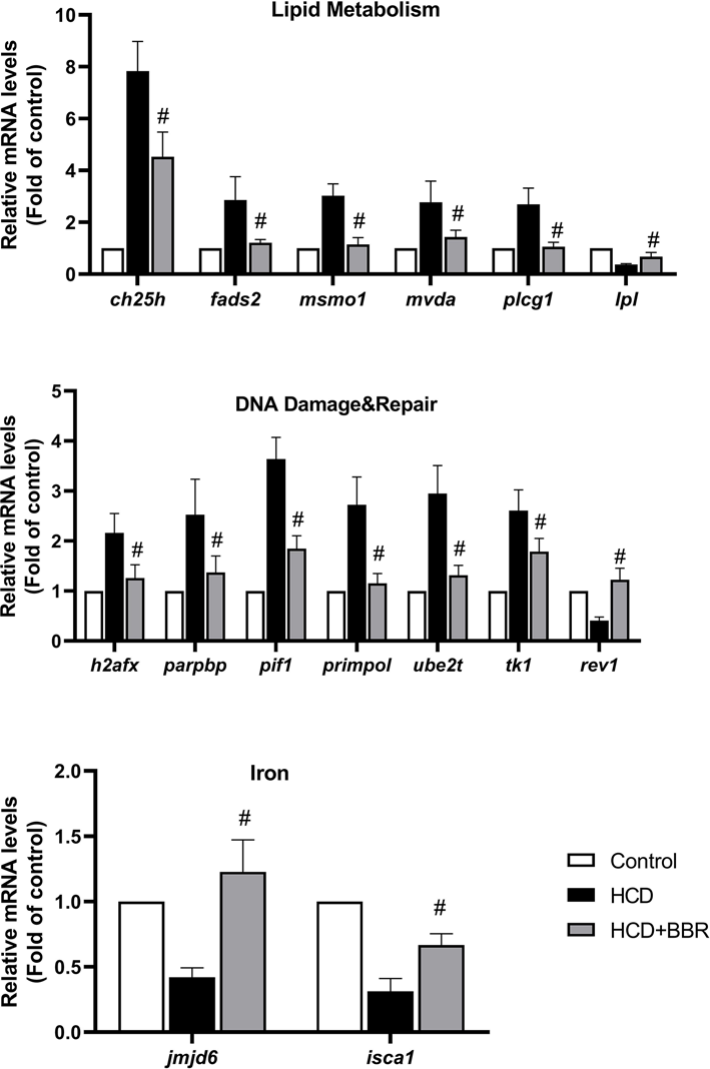
Validation of the microarray data using quantitative real-time PCR (qRT-PCR). Relative expression levels of the selected genes determined using qRT-PCR. ^#^Compared with the HCD group, *P* < 0.05.

To confirm the DEGs detected with microarray analysis, we selected the significant enrichment genes belonging to lipid metabolism, DNA damage and repair, and iron of which have the homologous gene in human for transcriptional validation (as shown in **Figure 7**). Results showed that BBR (5 μM) significantly decreased the gene expression, such as *ch25*, *fads2*, *msmo1*, *mvda*, *plcg1*, *h2afx*, *parpbp*, *pif1*, *primpol*, *ube2t*, and *tk1*, and increased the gene expression of *lpl*, *rev1*, *jmjd6*, and *isca1* (**Figure 7**). These gene expressions detected by qRT-PCR were in good agreement with those by microarray analysis.

## Discussion

NAFLD was commonly considered a disease of adults. However, accumulative evidence showed that the prevalence of NAFLD rise with the growing epidemic in the pediatric population around the world (Welsh et al., 2013). Several reports revealed that there is a heterogeneity in the patterns of the histologic lesions between pediatric and adult NAFLD/NASH (Crespo et al., 2016). This suggests that the pathophysiology of NAFLD may have significant differences between the adult and pediatric types (Papandreou et al., 2007). The pathogenesis of [ediatric NAFLD is still not fully understood. The scientific evidence indicates that a genetic predisposition interplaying with unhealthy diets and lifestyles might influence the occurrence and development of NAFLD in the pediatric population, mearly 50% of overweight children and adolescents accompanied by NAFLD (Betancourt-Garcia et al., 2017). In addition, insulin resistance and increased hepatic glucose production affect the hepatic *de novo* lipogenesis (Ciba and Widhalm, 2007). The knowledge gap of the pathogenesis of NAFLD/NASH in children is partially due to a lack of studies in animal models that may resemble pediatric NAFLD (Patton et al., 2006; Papandreou et al., 2007). In recent years, zebrafish have been gaining popularity in lipid research (Holtta-Vuori et al., 2010). We previously established a diet-induced steatosis model in larval zebrafish to mimic the progression of hepatic steatosis of the pediatric population and assess anti-steatosis agents (Chen et al., 2018). Given the transparent feature of larval zebrafish, we introduced Raman imaging and 3D molecular imaging to detect the change of the biochemistry composition and oxidative stress in zebrafish liver. These approaches could also be applied to other pharmacology and biochemistry study.

Several studies have reported that BBR exerts beneficial effects on NAFLD in adult patients and animal models through various potential mechanisms such as improvement of insulin sensitivity, adenosine monophosphate-activated protein kinase (AMPK) pathway, mitochondrial function, oxidative stress, and gut microenvironment (Yan et al., 2015; Zhu et al., 2016). The pediatric population is supposedly more susceptible to drug interventions (Hatton et al., 2018). Moreover, its availability and pharmacology mechanism may not be entirely consistent with adults (Hatton et al., 2018). Thus, we employed a diet-induced larval zebrafish model. Our results showed that BBR could significantly reduce the BMI, hepatic cholesterol, and triglyceride and improve hepatic steatosis in high-fat diet-treated zebrafish. These results suggest that berberine exerts its anti-steatosis through moderating the lipid metabolism and are similar to those obtained in adult rodent model studies (Zhao et al., 2017; Deng et al., 2019; Luo et al., 2019; Zhang et al., 2019). In addition, recent studies revealed that BBR could regulate adipose-mediated energy expenditure, which may exert an indirect effect on liver steatosis and further explain the reason for the reduction in body weight and BMI after berberine treatment in this study (Sun et al., 2018; Wu et al., 2019). Also, Raman mapping indicated that BBR treatment did not affect the hepatic biochemical composition in the healthy larvae. This evidence provides a new clue that BBR has the potential to prevent hepatic steatosis for the pediatric population.

Microarray is a high-throughput and rapid technique relative to traditional methods to detect transcriptome information. Accordingly, we investigated the transcriptome responses of zebrafish liver to BBR treatment using microarray. The transcriptome information of zebrafish liver under BBR treatment in the NAFLD model was presented for the first time and could provide the molecular basis for the pharmacological mechanism of BBR to larval zebrafish. GO analysis of the genes with differentially upregulated expression in the BBR group indicated that some genes were associated with the categories of response to virus (GO:0009615), antigen processing and presentation (GO:0019882), and innate immune response (GO:0045087). In addition to genetic and environmental factors on liver disease, increasing evidence showed that gut microbial composition has a strong influence on the pathogenesis of NAFLD (Bashiardes et al., 2016). Childhood allergic diseases exhibit high correlation to the early gut microbiota system in response to the immunity of infancy (Jenmalm, 2017). The changes in these associated immune genes might be supposed to high-fat diet challenge and BBR treatment. It has been reported that BBR could modulate hepatic lipid metabolism and exert hepatic protection by improving gut homeostasis (Sun et al., 2017; Qin et al., 2018; Shi et al., 2018). Thus, this may be a key mechanism of BBR in pediatric NAFLD treatment.

Moreover, GO term from differentially downregulated expression in the BBR group demonstrated that BBR might attenuate the fatty acid biosynthetic process, sterol biosynthetic process, and oxidation stress. KEGG pathway analysis suggested that the differentially expressed genes in the BBR group are mainly involved in the metabolic pathways, which are similar to previous reports (Qiang et al., 2016; Zhao et al., 2017; Deng et al., 2019). By functional annotation tool DAVID, we identified several essential genes involved in lipid metabolism, DNA damage and repair, and iron. Notably, studies showed that the prevalence of iron deficiency was higher in obese children than normal-weight subjects (Nead et al., 2004; Cepeda-Lopez et al., 2011). Moreover, children with NAFLD have higher serum levels of hepcidin than those without NAFLD (Amato et al., 2010). NAFLD may impact on iron homeostasis through a different mechanism from mere obesity-induced iron deficiency (Feldman et al., 2015). The regulation of iron metabolism by BBR needs further study and may become a potential approach for pediatric NAFLD. Besides, BBR has no effect on the genes related to liver lipogenesis (*srebp1*, *fas*, *acc1*, *acc2*, and *scd1*), fatty acid beta-oxidation (*ppara* and *cpt1a*), and VLDL secretion (*mtp*) in this study (**Figure S2**).

In recent years, Raman microspectroscopy has emerged as an attractive diagnostic tool (Zhu et al., 2016). Raman microspectroscopy provides a biochemical map of interested tissue that potentially enables the identification of spatial–temporal changes in molecular composition. Several studies reported the use of confocal Raman imaging for the detection of NAFLD (Hewitt et al., 2015; Yan et al., 2017). Raman imaging allows label-free approaches to observe and quantify the lipid accumulation within the liver without the need for contrast agents (Yan et al., 2017). Unlike rodent animal models, the biochemistry analysis of larval zebrafish liver is a challenge since the tiny size of the larval zebrafish liver is difficult to handle. We thus introduced Raman confocal microspectroscopy to analyze the changes of biochemistry component in the larval liver. Besides the differences in the lipid component, we also found an increase of DNA in the liver in HCD-treated zebrafish. Of note, NAFLD can ultimately lead to the development of hepatocellular cancer in some patients (Rinella, 2015). The increase in DNA may be indicative of malignancy (Cai et al., 2017). Particularly, BBR treatment could drop the peaks of DNA and lipid/protein, suggesting that it may delay the development of progressive NAFLD.

The pathogenesis of NAFLD and NASH remains poorly understood. The progression of NAFLD to NASH has been explained by the “two-hit” theory (Wruck et al., 2017). The “first hit” involves the accumulation of fat in the liver (Day and James, 1998). Oxidative stress and induction of adipocytokine are thought to play a role in the second “hit” (Spahis et al., 2017). It is convinced that NAFLD is closely associated with oxidative stress (Spahis et al., 2017). Excess lipids impact metabolic pathways, increase ROS generation, and damage to mitochondrial DNA (Dasarathy et al., 2011). TEM image showed that BBR corrected the abnormal structure of the mitochondria. Raman analysis suggested that BBR could affect DNA content. Molecular imaging demonstrated that BBR could eliminate hepatic ROS in the larval zebrafish model. By comprehensive analysis of those results, it indicated that BBR might alleviate excess ROS generation in the HCD-induced zebrafish model through improving mitochondrial quality. Cysteamine could reduce ROS by restoring glutathione levels (Caccamo et al., 2005). It acts as a novel therapeutic agent in nonalcoholic steatohepatitis (NASH), which has recently been used in a phase IIb trial in children with NASH (Schwimmer et al., 2016). The above evidence implies that BBR has the potential to treat pediatric NAFLD.

In summary, we first employed a larval zebrafish model to assess the effects and possible mechanism of BBR in the treatment of hepatic steatosis. Several genes involved in lipid metabolism, DNA damage and repair, and iron have been identified by microarray analysis. These results might provide molecular information that will facilitate understanding of BBR on the treatment of pediatric NAFLD. Also, further studies are required to validate and extend the findings of the results in this study. In research methods, based on the advantage of zebrafish, we introduced Raman imaging and 3D molecular imaging to analyze the biochemistry component and ROS level in the liver of zebrafish, respectively. Our study offers a new idea and extends the application of zebrafish models.

## Data Availability Statement

The datasets generated for this study can be found in the GEO repository, GSE136436, https://www.ncbi.nlm.nih.gov/geo/query/acc.cgi?acc=GSE136436.

## Ethics Statement

The animal study was reviewed and approved by the Laboratory Animal Management and Animal Welfare Committee in the Institute of Medicinal Biotechnology, Chinese Academy of Medical Sciences.

## Author Contributions

J-PZ conceived and designed this project. BC and J-PZ wrote the manuscript. C-QH reviewed the manuscript. BC performed the bioinformatics analysis and biochemistry experiment. Y-MZ and YH performed the Raman spectroscopy experiments, M-QZ performed the PCR experiment.

## Funding

This work was supported by the CAMS Initiative for Innovative Medicine (grant number 2016-I2M-1-011), by Foundation for Innovative Research Groups of the National Natural Science Foundation of China (grant number 81621064), and by the National Natural Science Foundation of China (grant number 81603172).

## Conflict of Interest

The authors declare that the research was conducted in the absence of any commercial or financial relationships that could be construed as a potential conflict of interest.
