# Supplementary figures and images for: Microarray Expression Profiling and Raman Spectroscopy Reveal Anti-Fatty Liver Action of Berberine in a Diet-Induced Larval Zebrafish Model
Source: Front Pharmacol. 2020 Jan 8;10:1504. doi: 10.3389/fphar.2019.01504 (PMC6960226; doi:10.3389/fphar.2019.01504)

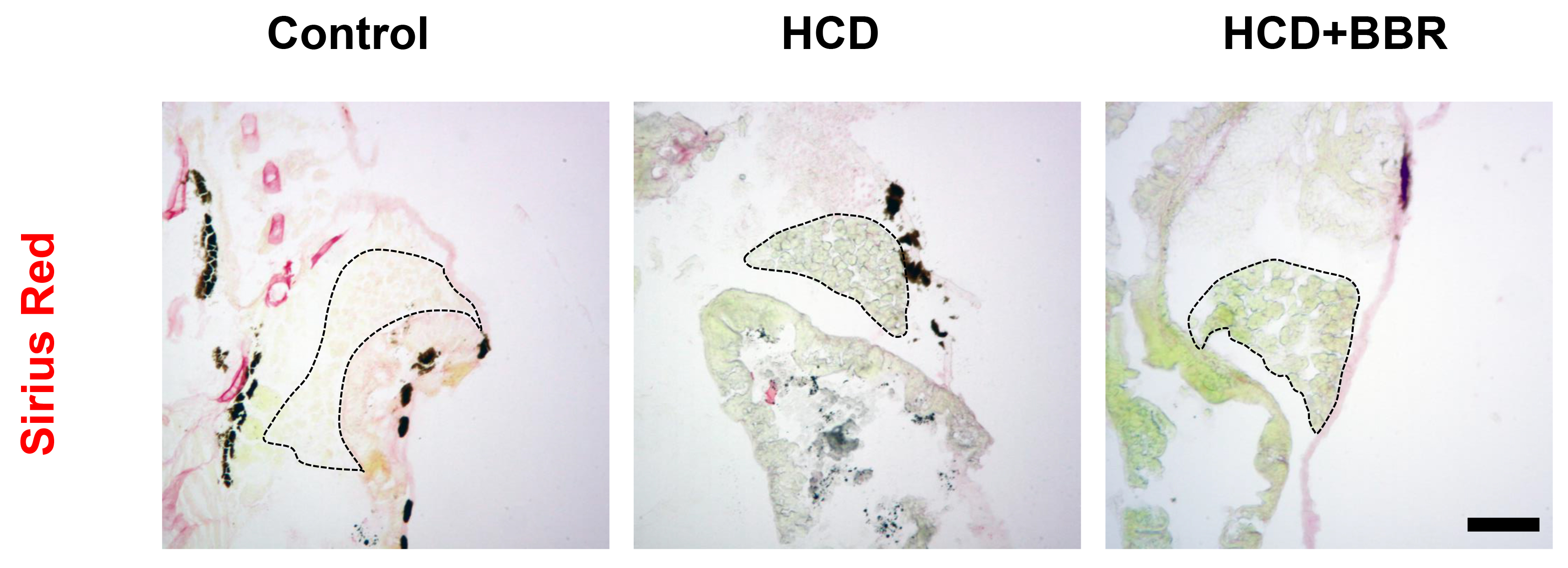

Supplement: Supplementary file 1 [file Image_1.jpeg]

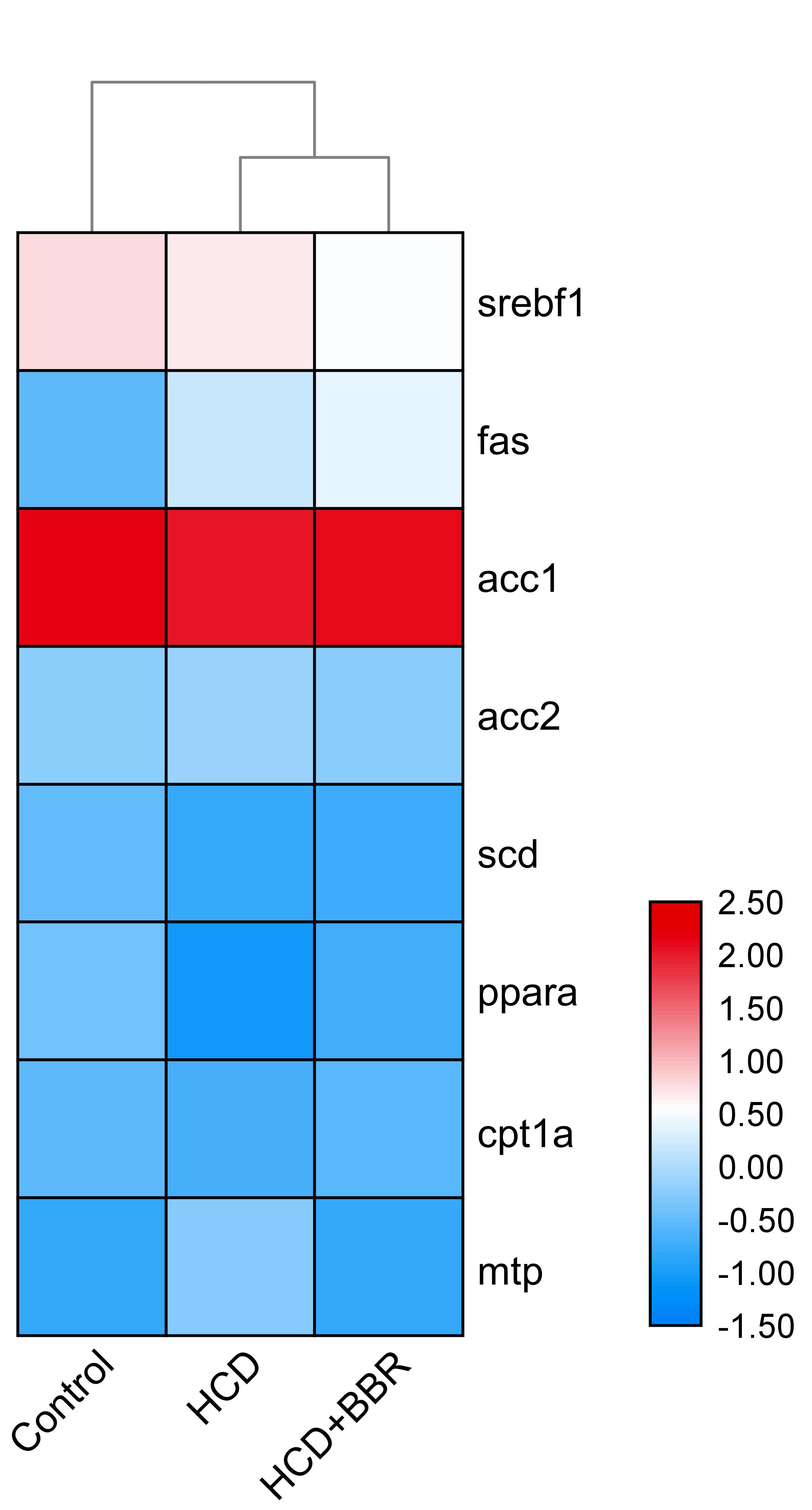

Supplement: Supplementary file 2 [file Image_2.jpeg]
